# Prevalence of Depressive Symptoms Among University Students in Pakistan: A Systematic Review and Meta-Analysis

**DOI:** 10.3389/fpubh.2020.603357

**Published:** 2021-01-08

**Authors:** Muhammad Naeem Khan, Parveen Akhtar, Saira Ijaz, Ahmed Waqas

**Affiliations:** ^1^Metro South Addiction and Mental Health Services, Brisbane, QLD, Australia; ^2^School of Medical Sciences, Griffith Health, Griffith University, Brisbane, QLD, Australia; ^3^School of Public Health, Global Health Institute, Xi'an Jiaotong University, Xi'an, China; ^4^Department of Psychiatry, King Edward Medical University, Lahore, Pakistan; ^5^Institute of Population Health, University of Liverpool, Liverpool, United Kingdom; ^6^Human Development Research Foundation (HDRF), Islamabad, Pakistan

**Keywords:** depression, university students, Pakistan, systematic review, meta-analysis (as topic), low resource setting

## Abstract

**Background:** In Pakistan, almost 30% population is between 15 to 29 years of age, with university enrollment rates of 10–15%. Although there is a growing concern on mental health of university students across the globe, studies from low and middle income countries such as Pakistan are scarce. We conducted a systematic review and meta-analysis of prevalence of depressive symptoms among Pakistani university students.

**Methods:** PubMed, Web of Science, PsycInfo and Google Scholar were searched between 15 to 30th January 2020. Cross-sectional and longitudinal studies, published till 31st December 2019 were included. Data on study characteristics and prevalence of depressive symptoms were extracted. Meta-analysis was conducted using random effects models. To estimate subgroup difference based on study characteristics, meta-regression and sub-group analyses were conducted.

**Results:** In total, 26 studies involving 7,652 participants were included in review. Overall prevalence of depressive symptoms was 42.66% (95% CI: 34.82% to 50.89%), with significant heterogeneity among studies. Subgroup analyses revealed a significant difference in prevalence estimates based on depression screening instrument and study major. Statistically significant differences were observed among studies employing different psychometric scales (test for subgroup differences, Q = 21.92, *p* < 0.05) and between students from different study majors (test for subgroup differences, Q = 3.76, *p* = 0.05).

**Conclusion:** Our study found that overall prevalence of depressive symptoms among university students in Pakistan was 42.66%, however, findings should be interpreted with caution. Large scale epidemiological surveys using valid and reliable tools are needed to better estimate prevalence of depression among Pakistani university students.

## Introduction

Depressive disorders are leading cause of disability worldwide (1, 2). Studies suggest that most Common Mental Disorders (CMDs) have their first onset before the age of 24 (3). Anxiety and mood disorders are highly prevalent among young people aged 18–29 years. Almost 40% of young people experience their first episode of depression before the age of 20, with an average age of onset in the mid-20s (4). These years are most important for education, employment and social relationships.

Over the last decade, there has been growing interest in the mental health of university students. Globally, 24 to 34% university students experience depressive symptoms (5–9). Depressive disorders are one of the major causes of years lost due to disability (YLDs) and Disability Adjusted Life Years (DALYs) in young people (10). Occurrence of depression during the critical period of transition from adolescence to adulthood may have adverse effects, not only on development and academic functioning, but also on future employment and work productivity. Studies have shown that depression leads to early attrition from university and poor academic performance (11–13). Moreover, depression is associated with lower employment prospects and unstable employment in adulthood (14).

## Pakistan-a Context

Pakistan is one of the youngest countries in the region, with almost 30% population between 15 to 29 years of age (15). In addition to having limited resources to invest in education and health, Pakistan has witnessed some major crises over the last two decades. The country was hit by a major earthquake in 2005 and heavy floods in 2010. A long wave of terrorism and militancy (2000–2014) did not even spare schools and colleges. More than 100 children were dead in a terrorists attack on Army public school Peshawar in 2014-the highest death tool in a single terrorist attack in the world. In 2016, a university in north-west province was attacked by terrorists, resulting in deaths of 19 students and teachers.

In Pakistan, a whole generation has grown up in an uncertain and insecure environment. Almost 70% population lives in rural areas. Meanwhile, over the last 20 years, trend of enrollment in higher education institutes has increased substantially with 10–15% of the eligible age group of 18–24 in universities or professional colleges (16, 17). Even from less privileged areas, young people are getting higher education. Most of these people are form the first generation of their families to receive higher education.

With almost non-existent career counseling and mental health services at campuses, university students in Pakistan battle with a highly competitive environment, financial constraints, future uncertainty and parental and societal demands to excel in studies and secure good jobs. All these stressors put university students at high risk of developing common mental health problems particularly depression. For a developing country like Pakistan, health and well-being of its youth is of utmost importance as they are the future human capital.

There is a need for reliable estimates of prevalence of mental health problems among university students to design interventions tailored to specific needs of youth in Pakistan. Present study aims to conduct systematic review and meta-analysis on prevalence of depression among university students in Pakistan.

## Methods

### Study Design

This systematic review and meta-analysis was done according to the Preferred Reporting Items for Systematic Reviews and Meta-Analyses (PRISMA) guidelines (18). A complete PRISMA Checklist is available as Supplementary Table 2.

### Participants, Intervention Comparators

Eligibility Criteria were defined according to the PICO (18).

**Population**- University students of any age.
**Intervention/Exposure**- Depression/depressive symptoms.
**Intervention**- not required for inclusion.
**Comparison**- not required for inclusion.

We included cross-sectional or longitudinal studies (baseline data) reporting prevalence of depressive symptoms among university students in Pakistan.

Exclusion criteria were studies reporting other study designs such as Randomized Controlled Trials (RCTs), case control studies, reviews (narrative and systematic), conference proceedings, case reports, qualitative studies, editorials, opinion papers, and letters. In addition, we did not include unpublished or non-peer reviewed articles.

### Systematic Review Protocol

Protocol for this systematic review is registered in International prospective register of systematic reviews (PROSPERO) under registration number CRD42020170099.

### Literature Search Strategy and Data Sources

We systematically searched PubMed, Web of Science, PsycInfo and Google scholar databases from January 15^th^ to 30^th^ 2020 for studies reporting primary data on depressive symptoms among university students in Pakistan, published till December 2019. In addition, the authors screened the reference lists of identified articles using the approaches implied by the Preferred Reporting Items for Systematic Reviews and Meta-analyses (18). For the database searches, a pre-tested search strategy, combining terms related to university students and depression was employed. To avoid irrelevant results, search was restricted to only English language studies as no research studies are published in local/national language of Pakistan (Complete details of the search strategy appear in Supplementary Table 1).

### Studies Selection and Data Extraction

The database searches were conducted by one author (MNK). After deletion of duplicate records using Endnote software, two authors (PA and MNK) independently screened all the titles and abstracts against the eligibility criteria. Any disagreements regarding inclusion for full-text screening were resolved through discussion with a third reviewer (SI). Thereafter, two authors (PA and MNK) independently reviewed the full-texts of all included articles. Disagreements were discussed with third author (SI) to achieve consensus. One author (PA) extracted data from all the included articles while 2nd author (MNK) extracted data from 25% of the studies to ensure accuracy and completeness of data extraction. Before starting the data extraction, both authors extracted data from three articles independently to establish inter-rater reliability. We found good inter-rater reliability between the two reviewers *(k* = 0.85).

Using a standardized data extraction sheet, data on following characteristics of included studies was extracted: author and publication years, study design, mean age of sample (or range, where mean was not available), sample size, sampling technique, number and percentage of females in the sample, education level, study major, instrument used to screen for depression, screening instrument cutoff, number of females with depression and overall prevalence of depressive symptoms.

### Risk of Bias

Risk of bias in the included studies was assessed using a modified version of the Joanna Briggs Institute (JBI) critical appraisal checklist for prevalence studies (19). JBI is frequently used quality assessment tool for prevalence studies (20–22). This checklist assesses each study on 9 items including sample representativeness, recruitment appropriateness, adequate sample size, description of subjects and setting, valid ascertainment and measurement of the condition, thoroughness of reporting statistical analysis, standard measurement for all participants and adequacy of response rate. We modified Item 5 (original item “Was data analysis conducted with sufficient coverage of the identified sample” changed to “was scale valid/reliable in Pakistani context). Studies were categorized to be at low risk of bias (≥7 points), moderate risk of bias (4–6 points) or high risk of bias (<4 points). The quality assessment did not determine inclusion/exclusion of the study in meta-analysis.

### Data Analysis

Descriptive statistics pertaining to prevalence of depressive symptoms and its overall severity were extracted. Studies were assessed based on methodological and statistical heterogeneity. Due to significant heterogeneity, data was pooled using random effects model and forest plots were generated displaying pooled prevalence with 95% confidence intervals. Between-study heterogeneity was assessed using standard χ^2^ tests, Tau^2^ and the I^2^ statistics (23, 24). I^2^ was presented as the percentage of variability in prevalence estimates due to heterogeneity rather than sampling error, or chance, with values ≥75% indicating considerable heterogeneity (23, 24). Sensitivity analysis using single study “knock out” approach was used to determine influence of each study on the pooled prevalence.

Publication bias was assessed by visual inspection of the funnel plot and Egger's tests (considered significant at *p* < 0.1). (25, 26). Duval and Tweedie's trim and fill method was used to adjust pooled prevalence estimate for publication bias (27). To explore heterogeneity among studies, we conducted subgroup analyses for categorical moderators, and meta-regression for continuous variables. Subgroups were conducted by field of study, level of education, university type (public /private), depression screening tool, sampling technique and study quality. All subgroup analyses were conducted using the mixed-effect method where a *p*-value of 0.05 was considered as having statistically significant subgroup differences.

Meta regression with maximum likelihood method and random effects was conducted to determine effect of age, sample size and percentage of females in the sample on the pooled prevalence. To ensure appropriate statistical power, we conducted subgroup analysis when subgroups were reported in at least four studies (28). While meta-regression analysis were run for moderators reported in at least ten studies (29). All the analysis were conducted in Comprehensive Meta-analysis software (CMA) version 3 (30). All statistical tests were 2-sided and *p*-values < 0.05 was considered statistically significant.

## Results

### Study Selection Process

**Figure 1:**
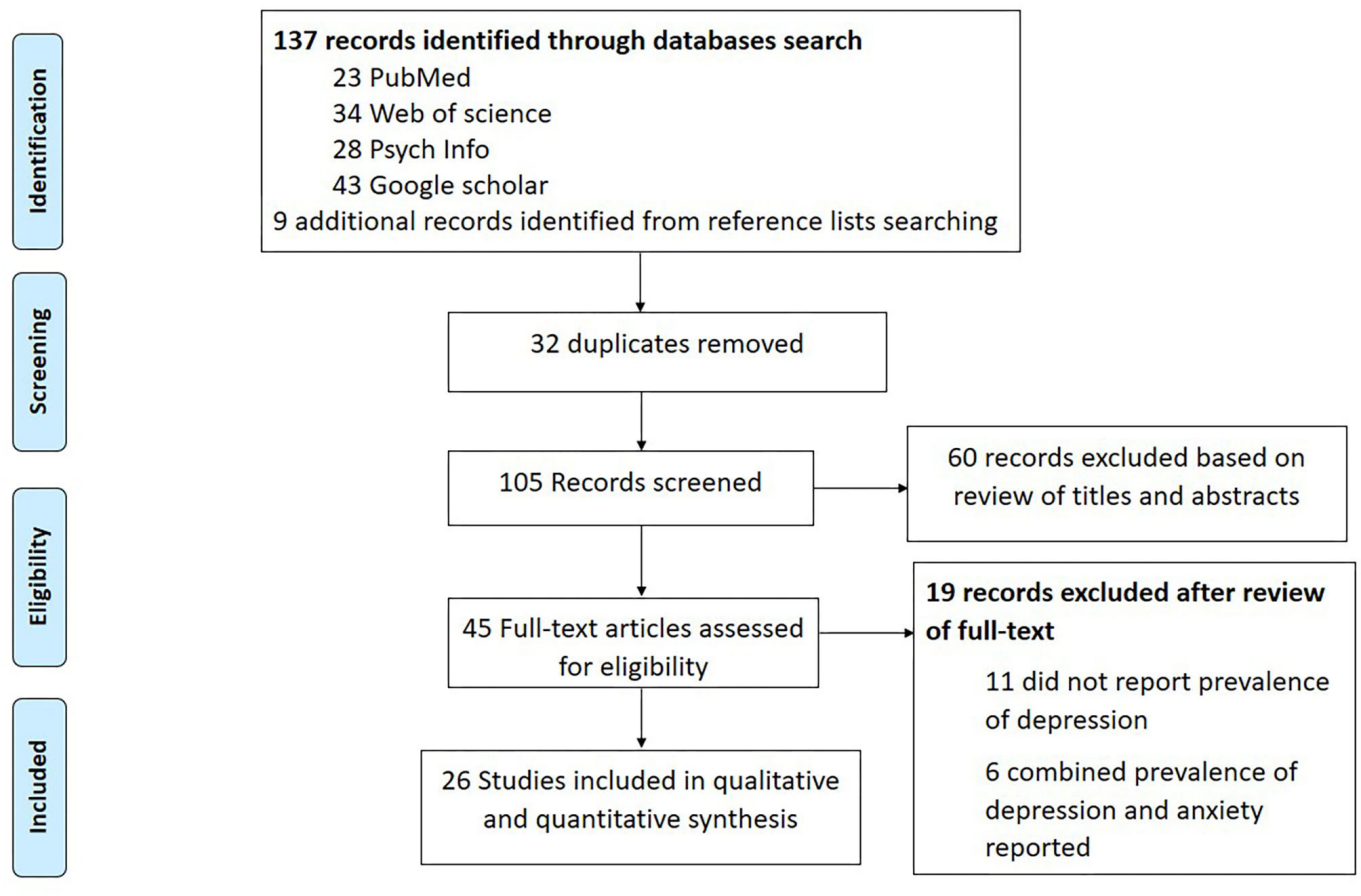
PRISMA flow diagram.

Our databases search yielded 137 records. After removal of 32 duplicates, 105 studies were screened for titles and abstracts against inclusion and exclusion criteria. After the screening process, a total of 45 full texts were found eligible for further assessment. We excluded 19 studies as the studies did not report prevalence of depression. A total of 26 full-texts were included in both the qualitative and quantitative synthesis. A detailed flow chart of the search and selection process is presented in Figure 1.

### Characteristics of Included Studies

Table 1 summarizes the basic characteristics of included studies. In total, 26 studies involving 7,652 participants were included in the analysis. The median number of participants per study was 289 (range, 66–1000). No longitudinal study was identified and all the included studies in our review were cross-sectional studies. Majority of the studies (24/26, 92%), were conducted with undergraduate students and only two studies included both undergraduate and graduate students. More than half (17/26, 65%) studies included only medical students. Among the studies conducted with students from non-medical majors, studies did not explicitly mentioned the study discipline. 10 (38.50%) studies recruited sample from public universities, 6 from private universities, 5 studies included mix sample from both, private and public universities, and 5 studies did not specify university type. Most of the studies used self-reporting screening tools to assess depression; 4 studies (15%) used Hospital Anxiety and Depression Scale (HADS), 3 studies (11.54%) used Beck Depression Inventory (BDI), 4 studies (15%) used Depression Anxiety Stress Scale-21 (DASS-21), 3 studies (11.54%) used Depression Anxiety Stress Scale-42 (DASS-42), Beck Depression Inventory-II (BDI-II), Center for Epidemiological Studies Scale for Depression (CESD) and Zungs Self-report Depression Scale (Zung-SDS) were used in two studies each. One study each used Quick Inventory for Depression Screen (QIDS), Patient Health Questionnaire-9 (PHQ-9), Duke Health Profile and Hamilton Depression Scale (HAM-D) while 3 studies did not specify the depression ascertainment methods.

**Table 1:** Characteristics of included studies.

	**References**	**Level of education**	**Field of study**	**University type**	**Depression screening instrument and cutoff**	**Sample size**	**Sampling technique**	**Age,** ** Mean (SD)**	**Females, No. (%)**
1.	Rizvi et al. (31)	Undergraduate	Medicine	Both public and private	DASS-42 ≥8	66	Non-random	22.15 (1.3)	38 (54.3)
2.	Khan and Ali (32)	Undergraduate	Nursing	Both public and private	BDI NR	150	Non-random	29.49 (5.67)	78 (52)
3.	Abbas et al. (33)	Undergraduate	Pharmacy	Not specified	NR	433	Non-random	Range 18-25 (21.5)	275(63.5)
4.	Bukhari and Khanam (34)	Mixed	NR	Not specified	CES-D≥16	331	Non-random	21.70 (2.7)	166 (50)
5.	Alvi et al. (35)	Undergraduate	Medicine	Private	BDI-II ≥14	279	Non-random	21.4(1.41)	202 (72.4)
6.	Zafar et al. (36)	Undergraduate	Medicine	Both public and private	BDI-II≥20	300	Non-random	23 (2)	218 (72.7)
7.	Naz et al. (37)	Undergraduate	Medicine	Private	DASS-21 ≥10	129	Non-random	19 (17–21)	88(68)
8.	Bibi et al. (38)	Mixed	Multiple, NR	Public	BDI NR	436	Non-random	24.32	196 (45)
9.	Ikram et al. (39)	Undergraduate	Medicine	Public	CES-D≥16	154	Non-random	21.10 (1.93)	93 (60.4)
10.	Marwat (40)	Undergraduate	Medicine	Public	(Zung-SDS ≥51	166	Non-random	NR	NR
11.	Chaudhry et al. (41)	Undergraduate	Medicine	Private	HADS-D ≥8	250	Non-random	NR	125 (50)
12.	Gitay et al. (42)	Undergraduate	Medicine	Public	PHQ-9 ≥5	300	Non-random	NR	185 (61.7)
13.	Saeed et al. (43)	Undergraduate	NR	Both public and private	DASS-42 ≥14	404	Non-random	NR	211
14.	Haq et al. (44)	Undergraduate	NR	Public	DASS-21 ≥14	361	Random	NR	191 (52.9)
15.	Buzdar et al. (45)	Undergraduate	Social sciences	Not specified	DASS-21 NR	600	Random	NR	502 (100)
16.	Kumar et al. (46)	Undergraduate	Medicine	Both public and private	DASS-21 ≥10	312	Non-random	22.7 (1.52)	264 (84.6)
17.	Syed et al. (47)	Undergraduate	Physiotherapy	Not specified	DASS-42 NR	267	Non-random	19.3 (1.19)	201 (75.3)
18.	Ghayas et al. (48)	Undergraduate	NR	Not specified	Zung-SDS ≥50	408	Non-random	21.36(2.594)	248; 60.8
19.	Waris et al. (49)	Undergraduate	Medicine	Public	Duke Health Profile ≥25	300	Non-random	Range 18-25 (21.5)	NR
20.	Azad et al. (50)	Undergraduate	Medicine	Private	BDI ≥14	150	Non-random	20.6(0.88)	112 (74.6)
21.	Rehman et al. (51)	Undergraduate	Medicine	Public	Predesigned Questionnaire NR	150	Non-random	18-26 (22)	75,50%
22.	Aziz et al. (52)	Undergraduate	Medicine	Public	HAM-D ≥10	100	Non-random	NR	50 (50)
23.	Rab et al. (53)	Undergraduate	Medicine	Public	HADS-D ≥8	87	Random	20.7 (1.9)	NR
24.	Perveen et al. (54)	Undergraduate	Medicine	Public	QIDS ≥10	1000	Non-random	NR	569 (56.9)
25.	Khan et al. (55)	Undergraduate	Medicine	Private	HADS-D ≥11	110	Non-random	21	55 (50)
26.	Waqas et al. (56)	Undergraduate	Medicine	Private	HADS-D ≥8	409	Random	19.9 (1.33)	286 (70%)

*BDI, Beck Depression Inventory; CES-D, Center for Epidemiological Studies Depression Scale; DASS, Depression Anxiety Stress Scale; HAM-D, Hamilton depression scale; HADS-D, Hospital Anxiety and Depression Scale; NR, not reported; PHQ-9, 9-item Patient Health Questionnaire; QIDS, Quick Inventory for Depression Screen; Zung-SDS, Zung Self-Rating Depression Scale*.

### Synthesized Findings

#### Prevalence of Depression in University Students in Pakistan

**Figure 2:**
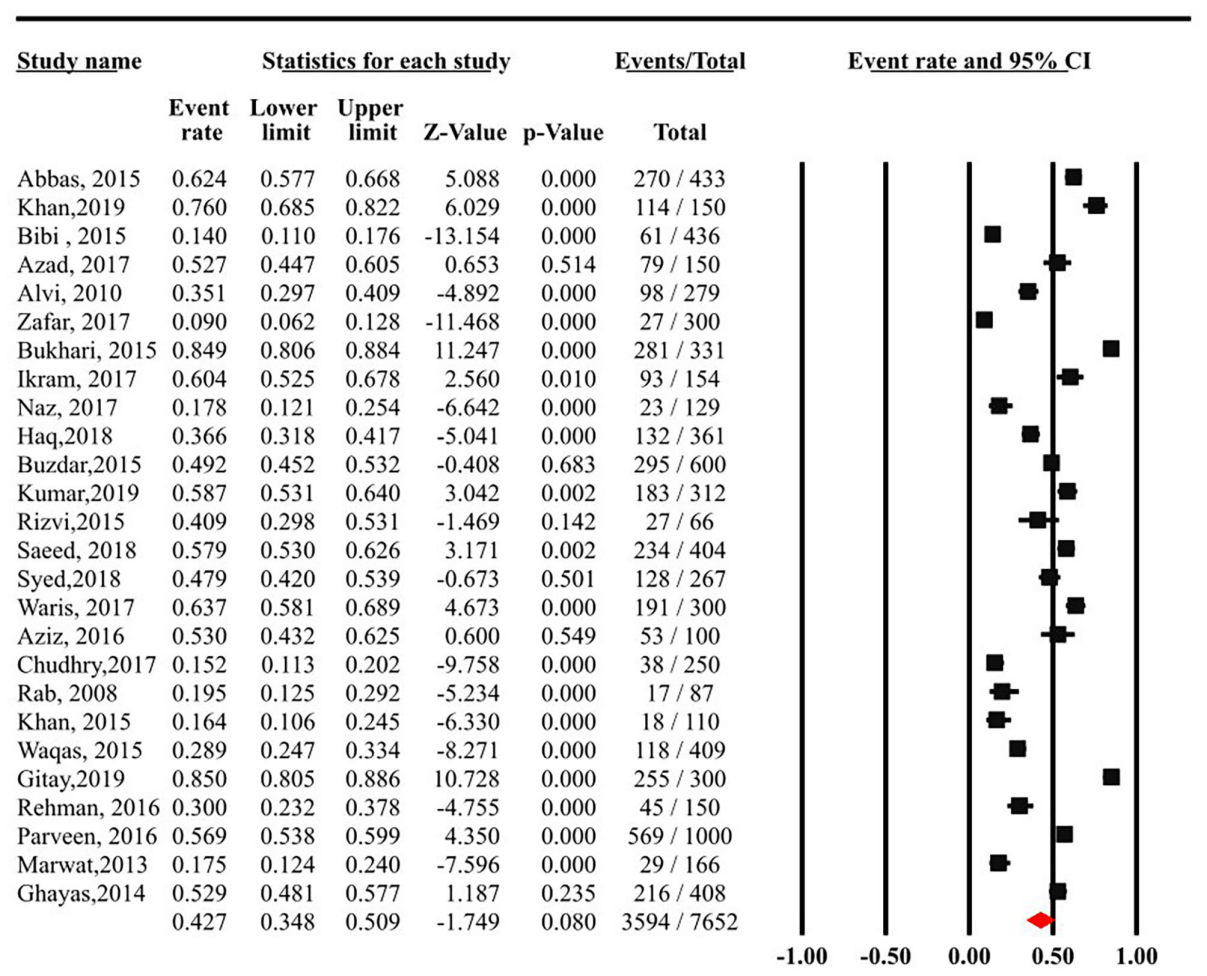
Meta-analysis of 26 studies on prevalence of depressive symptoms among university students in Pakistan.

There was an evidence of substantial statistical heterogeneity among the included studies (I^2^= 97.68%, Cochran's Q = 1078.55, *p* < 0.001). Therefore, random effects were employed while pooling event rates across studies, yielding a pooled prevalence rate of 42.66% (95% CI: 34.82 to 50.89%) (see Figure 2). Out of 7,652 university students, a total of 3,549 reported having depressive symptoms according to different screening tools.

#### Subgroup Analyses

Several subgroup analyses were conducted in this meta-analytical investigation. Prevalence of depression among undergraduate students (*n* = 24) was slightly lower as compared to studies that included sample from both graduate and undergraduate levels (*n* = 2). Among undergraduates students a prevalence rate of 42.24% (95%CI: 33.5-49.79%) of depressive symptoms was reported as compared to 48.86% (95% CI:2.88-96.85%) by other student population. Studies employing random sampling yielded lower prevalence rates (33.47%, 95% CI: 23.26-45.51%) than non-random counterparts (44.49%, 95% CI: 35.29-54.09%). Studies with lowest risk of bias reported the lowest prevalence rate of 30% (95% CI: 31.13-51.37%) than their counterparts with moderate (48.09%, 95% CI: 36.53-59.86%) and highest risk of bias (40.71%, 95% CI: 14.82-51.37%), however, none of these difference was statistically significant (Table 2A).

**Table 2A:** Subgroup analysis based on study characteristics.

**Group**	**Sub-groups**	**Number of studies**	**Prevalence (%)**	**95% CI**	**I^**2**^**	**Q**	**df**	**P**
Study level	Mixed	2	48.86	2.88-96.85	99.66	0.02	1	0.88
	Undergraduate	24	42.24	35.04-49.79	97.056			
Sampling technique	Non-random	22	44.49	35.29-54.09	97.81	2.06	1	0.15
	Random	4	33.47	23.26-45.51	94.71			
Risk of bias	High	5	30.00	14.82-51.37	96.69	2.38	2	0.30
	Low	6	40.71	31.13-51.06	95.57			
	Moderate	15	48.09	36.53-59.86	97.95			
Study discipline	Medicine	17	36.90	27.14-47.86	97.57	3.76	1	<0.05
	Other	9	53.59	40.71-66.01	97.98			
University type	Public	10	42.60%	29.45-56.90	98.07	3.13	2	0.21
	Private	5	26.13%	42.71-93.76	93.76			
	Both public and private^*^	6	5.94%	27.31-65.77	97.85			

* *Studies recruited sample from both public and private universities*.

Students enrolled in disciplines other than medicine reported higher prevalence of depressive symptoms (53.59%, 95% CI: 40.71%-66%) as compared to medical students (36.90%, 95% CI: 27.14-47.86%). The difference was statistically significant (test for subgroup differences, Q = 3.76, *p* = 0.05). Lowest percentage of depressive symptoms were reported by private sector university students (26.13%; 95% CI: 14.37-42.71%) than those studying in public (government funded) universities (42.60%, 95% CI: 29.45-56.90) or studies which included sample from both public and private universities (45.94% 95%CI: 27.31-65.77%). However, this difference did not yield statistical significance (test for subgroup differences, Q = 3.13, *p* = 0.21) (see Table 2A).

When comparing prevalence rates of depression between studies employing different psychometric scales, statistically significant differences were observed (test for subgroup differences, Q = 21.92, *p* < 0.05). There was evidence of significant variation in the extent of heterogeneity observed across studies employing different scales. Lowest prevalence of depressive symptoms was reported as per BDI-II scale and the highest according to CES-D and HAM-D scale (see Table 2B).

**Table 2B:** Subgroup analysis based on depression screening instrument.

**Sub-group**	**Number of studies**	**Prevalence (%)**	**95% CI**	**I^**2**^**	**Q**	**df**	***p***
BDI	3	45.06	24.41-67.57	98.88	21.92	9.00	<0.05
BDI-II	2	19.03	6.98-42.42	98.05			
CES-D	2	74.57	48.39-90.17	97.02			
DASS-21	4	39.55	22.66-59.37	95.73			
DASS-42	3	49.12	27.52-71.05	80.83			
HADS-D	4	19.61	9.72-35.59	84.64			
HAM-D	1	53.00	18.10-85.20	0.00			
Zung-SDS	2	33.23	13.73-60.88	98.15			
Other	4	60.67	40.88-77.48	97.55			

*BDI, Beck Depression Inventory; CES-D, Center for Epidemiological Studies Depression Scale; DASS, Depression Anxiety Stress Scale; HAM-D, Hamilton depression scale; HADS-D, Hospital Anxiety and Depression Scale; NR, not reported; PHQ-9, 9-item Patient Health Questionnaire; QIDS, Quick Inventory for Depression Screen; Zung-SDS, Zung Self-Rating Depression Scale*.

#### Meta Regression Analysis

Meta-regression analyses using random effects model was conducted to analyze association between prevalence rates of depressive symptoms, age of sample, total sample size and proportion of females in the sample. Each variable accounted for only 3% of variance in heterogeneity in the reported effect size, and did not yield statistical significance (*p* > 0.05). (see Tables 3A–C).

**Table 3A:** Meta-regression analysis for the prevalence (%) of depression in university students with proportion of females.

**Covariate**	**Coefficient**	**S.E**	**95% CI**	**Z-value**	***P***
Intercept	0.9801	2.0903	−3.11-5.07	0.47	0.64
Proportion of females	−0.0179	0.0323	−0.08-0.04	−0.55	0.58

*R^2^ = 3%*.

**Table 3B:** Meta-Regression analysis for the prevalence (%) of depression in university students with mean age of sample.

**Covariate**	**Coefficient**	**S.E**	**95% CI**	**Z-value**	***p***
Intercept	−2.1153	3.0972	−8.18-3.95	−0.68	0.49
Mean age	0.0872	0.1375	−0.18-0.36	0.63	0.52

*R^2^ = 3%*.

**Table 3C:** Meta-regression analysis for the prevalence (%) of depression in university students with sample size.

**Covariate**	**Coefficient**	**S.E**	**95% CI**	**Z-value**	***p***
Intercept	−0.61	0.38	−1.35-0.12	−1.63	0.1
Sample size	0	0	0-0	0.9	0.37

*R^2^ = 3%*.

#### Sensitivity Analysis

Sensitivity analysis did not indicate any changes in the mean prevalence when individual studies were removed from the meta-analysis, except the removal of two studies (34, 42) independently reduced the prevalence rate of depression from 42.7 to 40%. (See Supplementary Figure 1).

#### Assessments of Publication Bias

There was some evidence of publication bias in reporting of prevalence of depression among university students (Egger's statistic = −6.09 (3.44), *p* = 0.09).

**Figure 3:**
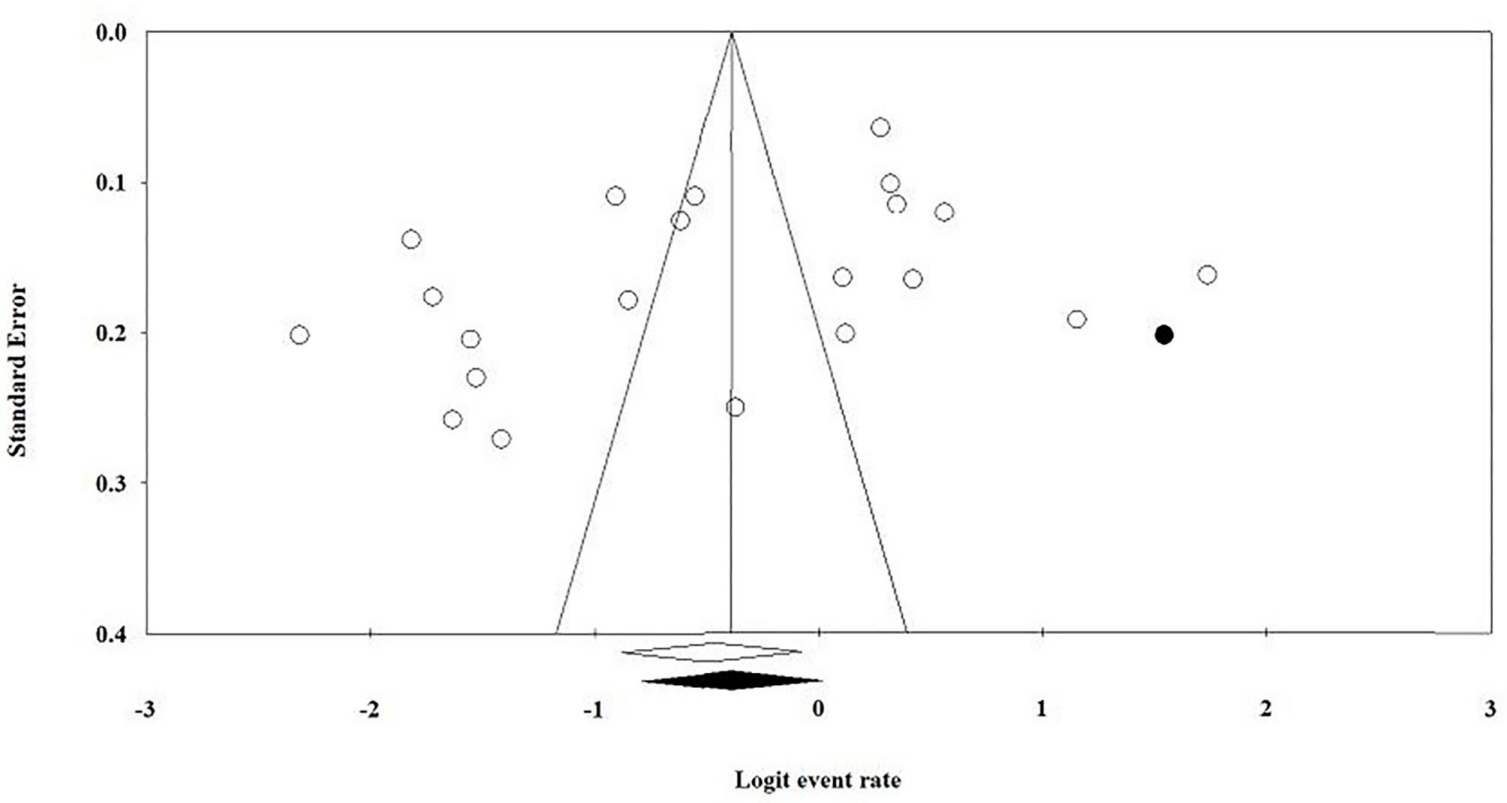
Funnel plot for publication bias with trim and fill method.

Trim and fill method using random effects was used to adjust the pooled prevalence estimates for publication bias. After imputing one study to the right of mean, it yielded an adjusted prevalence of 40.45% among university students (95% CI: 31.21% to 50.42%) (see Figure 3).

#### Risk of Bias

Most of the included studies had a moderate to high risk of bias. Mean quality score was 5.12 (SD; 1.53) out of 9. Only 6 studies had low risk of bias, while 15 out of 26 (58%) studies had a moderate to high risk of bias. Out of 26 studies, 21 studies did not report or cite the reference of scale's psychometric properties for Pakistani population. Only 4 (15%) studies employed random sampling technique, and 11 (42%) studies included sample from multiple schools/universities. Response rate was given in 12 (46%) studies. Risk of bias score for all individual studies has been shown in Table 4.

**Table 4:** Risk of bias in included studies.

	**References**	**Sample representativeness**	**Appropriate sampling technique**	**Sample size**	**Description of study sample**	**Scale reliable and valid**	**Ascertainment of depression**	**Standard measurement**	**Appropriate statistical analysis**	**Response rate**	**Total score**	**Risk of bias**
1.	Rizvi et al. (31)	0	0	0	0	0	1	1	1	0	3	High
2.	Khan and Ali (32)	1	0	0	1	0	0	1	1	0	4	Moderate
3.	Abbas et al. (33)	1	0	1	1	0	0	1	1	1	6	Moderate
4.	Bukhari and Khanam (34)	1	0	1	0	0	1	1	1	0	5	Moderate
5.	Alvi et al. (35)	0	0	1	1	0	1	1	1	1	6	Moderate
6.	Zafar et al. (36)	1	0	1	1	0	1	1	1	0	6	Moderate
7.	Naz et al. (37)	1	0	0	1	0	1	1	1	1	6	Moderate
8.	Bibi et al. (38)	0	0	1	0	0	0	1	1	0	3	High
9.	Ikram et al. (39)	0	0	0	0	0	1	1	1	0	3	High
10.	Marwat et al. (40)	0	0	0	0	0	1	1	1	1	4	Moderate
11.	Chaudhry et al. (41)	0	0	1	0	0	1	1	1	1	5	Moderate
12.	Gitay et al. (42)	0	0	1	0	0	1	1	1	0	4	Moderate
13.	Saeed et al. (43)	1	0	1	1	0	1	1	1	1	7	Low
14.	Haq et al. (44)	0	1	1	1	1	1	1	1	0	7	Low
15.	Buzdar et al. (45)	1	1	1	0	1	1	1	1	1	8	Low
16.	Kumar et al. (46)	1	0	1	0	0	1	1	1	1	6	Moderate
17.	Syed et al. (47)	0	0	1	1	0	1	1	1	0	5	Moderate
18.	Ghayas et al. (48)	1	0	1	1	1	1	1	1	0	7	Low
19.	Waris et al. (49)	0	0	1	0	0	1	1	1	0	4	Moderate
20.	Azad et al. (50)	0	0	0	1	0	1	1	1	1	5	Moderate
21.	Rehman et al. (51)	0	0	0	1	0	0	1	1	0	3	High
22.	Aziz et al. (52)	0	0	0	0	0	1	1	1	1	4	Moderate
23.	Rab et al. (53)	0	1	0	1	1	1	1	1	1	7	Low
24.	Perveen et al. (54)	1	0	1	0	0	1	1	1	0	5	Moderate
25.	Khan et al. (55)	0	0	0	0	0	1	1	1	0	3	High
26.	Waqas et al. (56)	1	1	1	0	1	1	1	1	1	7	Low

## Discussion

### Summary of Main Findings

In this systematic review and meta-analysis of 26 studies involving 7652 university students, prevalence of depressive symptoms was found to be 42.66% (95% CI: 34.8-50.9%). Overall prevalence is higher than the recent estimated prevalence rates of 24% (95% CI, 19.2%−30.5%) among university students in LMICs as reported by Akhtar et al. (9) as well as recent global estimates among medical students (27%, 95% CI, 24.7 to 29.9%) reported by Rotenstein et al. (7). This is alarming given relatively low university enrollments rates in low resource countries like Pakistan.

University environment in Pakistan is getting more and more competitive. University teachers, parents and society in general value high achievers. The constant pressure of getting good grades and landing a decent job may lead to feeling of stress and depression. There are no psychological and career counseling services at university campuses. Very few available metal health services are concentrated in tertiary healthcare facilities in big cities. In addition, lack of awareness and training among teachers to recognize and support students with common mental health problems and stigma attached to mental health problems are major barriers in seeking professional help. All these factors cause unnecessary delay in treatment, resulting in worsening the problems.

Prevalence of depression among students with non-medical majors was significantly higher than those with medical. Those enrolled in medicine reported lower prevalence of depression (36.90%, 95% CIs: 27.14-47.86%) than those in degree programs other than medicine (53.59%, 95% CI: 40.71-66%). In Pakistan, medicine and engineering are the first choice of most of students and their parents. However, securing admission in these fields is very competitive due to limited number of public medical and engineering colleges. Many students who cannot make to medical and engineering colleges, choose other fields. At one hand, they may feel less satisfied and not being able to fulfill the expectations of parents, and frustrated with highly competitive job market and limited career opportunities on the other hand. However, it should be noted that there were very few studies having sample from non-medical study majors in this review.

Significant difference in prevalence estimates was found in studies using different screening tools. Different tools employ different cut-offs and sometimes same tool can be used with different cut-offs. Moreover, most of studies did not mention the psychometric properties for Pakistan population. Previous studies also indicate a difference in prevalence estimates based on screening instruments (7).

Quality assessment of studies indicated few high quality studies. Only few studies employed random sampling techniques and recruited sample from multiple school, this could have introduce a selection bias in the individual studies included in this review, indicating scarcity of large scale, valid and reliable surveys.

Moreover, we found only 26 studies in four major databases, without publication dates restrictions. This is an indication of overall scarcity of research in this field in Pakistan

A high prevalence of depressive symptoms among Pakistani university students is a threat to healthy development of students and their smooth transition to adulthood. It may have long-term adverse effects for individuals as well the nation. Researchers and policy maker should focus this problem in future research. There is need for valid and reliable estimates prevalence of depressive symptoms among Pakistani university students, following guidelines for large epidemiological studies (57). Longitudinal studies are needed to analyze risk and protective factors for depression, with a focus on cultural factors. Barriers to access to mental health services need to be addressed by campus-based mental health services and community based interventions to reduce stigma associated with mental health problems. Due to the socio-political situation in general and in the specific context of COVID-19 outbreak, there is a need to integrate psychological wellbeing strategies in the university curricula. This will help students to combat the adversities they are constantly exposed to as well as serve as a solution to scarcity of specialized and community-based mental health services. Teachers training in identification and recognition of common mental health disorders, and basic counseling skills can also be integrated in usual teachers training.

## Strengths and Limitations

This is the first study to systematically review the prevalence of depression among university students in Pakistan. We conducted meta-analysis to summarize prevalence estimates. We did not apply any restrictions on publication date to include as many studies as possible.

Our findings should be interpreted under the light of a few limitations. Studies included in this review used variety of screening tools, different sample sizes and screening tools cut-offs, that introduced substantial heterogeneity. Depression ascertainment methods employed by the most of studies in our systematic review were self-reporting screening tools. These tools do not provide clinical diagnosis. Most of studies did not report the psychometric properties for Pakistani population. We did not included gray literature such as non-published or non-peer reviewed studies in our meta-analysis, which may have introduced publication bias in present results. One more limitation of the current review is that we did not include any social factors for depression or co-morbidities in our analyses.

## Conclusion

In this systematic review and meta-analysis, prevalence of depressive symptoms among Pakistani university students was found to be 42.66% with a huge variation among studies, however, there were very few good quality studies. Future research efforts should be directed to conduct large epidemiological studies for valid and reliable estimates of depression and to implement interventions to prevent and treat depression among university students.

## Data Availability Statement

The original contributions presented in the study are included in the article/Supplementary Material, further inquiries can be directed to the corresponding author/s.

## Author Contributions

MNK and PA conceptualized and designed the study. PA, SI, and MNK performed the article search and data extraction. AW and MNK analyzed the data. AW, MNK, and PA interpreted the results. MNK and PA drafted the manuscript in support with AW and SI. All authors reviewed and approved the final version of the manuscript.

## Conflict of Interest

The authors declare that the research was conducted in the absence of any commercial or financial relationships that could be construed as a potential conflict of interest. The reviewer SS declared a shared affiliation, though no other collaboration, with one of the authors AW to the handling Editor.
